# Surface Properties of Coatings Based on Iron Amino-Functionalized Oxides Deposited on DH 36 Steel Plates for Shipbuilding

**DOI:** 10.3390/nano15030150

**Published:** 2025-01-21

**Authors:** Maria Luisa Testa, Carla Calabrese, Valeria La Parola, Cristina Scolaro, Annamaria Visco, Simone Cappello, Leonarda Francesca Liotta

**Affiliations:** 1 Institute for the Study of Nanostructured Materials (ISMN)—CNR, via Ugo La Malfa, 153, 90146 Palermo, Italy; marialuisa.testa@cnr.it (M.L.T.); carla.calabrese@cnr.it (C.C.); valeria.laparola@cnr.it (V.L.P.); 2 Department of Engineering, University of Messina, C. da Di Dio, 98166 Messina, Italy; 3 Institute for Polymers, Composites and Biomaterials—CNR IPCB, Via Paolo Gaifami 18, 95126 Catania, Italy; 4 IRBIM-CNR, Spianata San Rainieri, 86, 98122 Messina, Italy; simone.cappello@irbim.cnr.it

**Keywords:** iron amino-functionalized oxide, steel for shipbuilding, coating, adhesion test, roughness

## Abstract

The development of eco-friendly paint formulations is part of the transition process to more sustainable materials, which involves many industries such as offshore and shipbuilding, where the deterioration of steel in seawater is a key factor. This article aims to produce innovative coatings and test their protective action on DH 36 steel plates. SiO_2_ and TiO_2_ were modified with amino groups and iron sites to be used as filler for the design of ecological paint formulations The antimicrobial features of both NH_2_ groups and iron ionic species were combined with the chemical and mechanical stability of silica and titania, with silica-based powders showing increased efficacy. The surface properties of the resulting coatings were examined by determination of thickness, water wettability, roughness, and cross-cut adhesion tests (before and after a degradation test in seawater according to ASTM D870-97 standards). Preliminary tests of the microbiological activity of the iron amino functionalized materials were carried out to monitor, as proof of concept, the growth of some bacterial strains through measurements of optical density. The findings indicate that these coatings not only provide effective corrosion protection but are promising for enhancing the durability and environmental performance of steel surfaces exposed to marine environments.

## 1. Introduction

The development of more sustainable solutions to environmental concerns is a common goal that encompasses materials science technologies. Among them, paint and coating systems focus on the use of less toxic components while preserving high physical/chemical and mechanical properties [1,2,3]. This need is particularly felt in shipbuilding, since the steel used for hull construction is subject to corrosion in seawater and biofouling while also having to meet the requirement to operate at extremely low temperatures [4].

A suitable strategy to enhance the functional features of organic coatings entails the addition of nanomaterials such as SiO_2_, TiO_2_, ZnO, Al_2_O_3_, CaCO_3_, and CeO_2_. The incorporation of targeted nanopowders into a polymeric matrix may influence the coating performance and optimize the mechanical, chemical, and optical properties [5]. Some studies reported the functional performance of nanoparticles of SiO_2_, Zn, and Fe_2_O_3_ as anti-corrosive agents as well as the mechanical properties of modifiers for epoxy [6] and silane-based sol–gel [7] coatings. The peculiar properties of nanoparticles have been utilized in the coating industry to improve the physical, mechanical, and chemical properties of organic coatings [8,9,10,11].

A great emphasis is placed on the utilization of easily available starting materials such as commercial silica gel, whose high specific surface area combined with its high thermal and chemical stability allows a wide range of surface modifications. Another example of a commercial nanopowder for the preparation of functional paints and coatings is titanium dioxide, which emerged in the field of photocatalysis for environmental applications [12]. The demand for effective solutions to address marine biofouling challenges has driven the advancement of antifouling and anticorrosion coatings, often incorporating nanocomposite materials with biocidal agents or utilizing the fouling release mechanism [13,14,15,16,17,18]. More broadly, the development of nanocomposite materials with antimicrobial and antifouling characteristics is a widely studied area, with applications across diverse research domains, including the creation of biomedical devices, water purification systems, food packaging, and marine equipment. The biocide action of some metal and metal oxide nanoparticles (NPs), in particular silver (Ag) NPs, copper (CuO), and zinc oxide (ZnO), is well known [19,20]. The studies on these materials are still increasing due to their broad-spectrum antimicrobial properties.

In general, they can act by adhesion onto the bacterial surface followed by the destabilization of the cell wall, inducing modifications of membrane permeability or causing toxicity and oxidative stress by the generation of reactive oxygen species (ROS). Recently, EuChems (www.euchems.eu) https://www.euchems.eu/euchems-periodic-table/ (accessed on 17 January 2025) highlighted the issue of element scarcity arising from limited supplies, their locations in conflict areas, or the inability to fully recycle them. Despite their effective biocide activity, these elements are classified as posing a serious threat in the next 100 years (Ag and Zn), with limited availability and future risk to supply (Cu). Conversely, iron (Fe) belongs to those elements with a plentiful supply. Moreover, iron-containing systems have shown antimicrobial features [19,21] acting in the form of iron cations [22,23,24], generating ROS, and finally, leading to damage to the cell membrane. Thanks to their physical/chemical properties, biocompatibility, and plentiful supply, iron-based materials could be good candidates for designing sustainable materials. Furthermore, studies in the literature report the application of materials containing quaternary ammonium salts [25,26] and —NH_2_ moieties [27,28,29,30,31] with antibacterial and antifouling activity against a variety of microorganisms (such as *Staphylococcus aureus*, *Escherichia coli*, *Candida albicans*). Recently, some of us have described the excellent antibacterial properties of aminopropyl waste cellulose-derived materials [32,33]. In these studies, cellulose and lignocellulose supports were functionalized with (3-aminopropyl)triethoxysilane (APTES) in sustainable reaction conditions. The antibacterial activity against Gram-positive and Gram-negative bacteria was then evaluated both in batch [32] and in continuous flow [33], showing high bacteria removal (93–100%). Considering these premises, the present work aims to combine the antimicrobial features of both NH_2_ groups and iron ionic species with the chemical and mechanical stability of commercial silica and titania, using them as solid supports to obtain advanced materials for the development of more sustainable coatings with potential antifouling applications. We easily prepared two organic/inorganic hybrid nanopowders by grafting 3-aminopropyl triethoxysilane (APTES) onto the surface of SiO_2_ and TiO_2_ bare supports. The resulting samples (SiO_2_-NH_2_ and TiO_2_-NH_2_) were, in turn, impregnated with iron ionic species.

The final materials (SiO_2_-NH_2_-Fe and TiO_2_-NH_2_-Fe) were then characterized and employed for the preparation of paint formulations which were applied to the surface of metal prototypes.

The surface properties of the resulting coatings, deposited on DH 36 steel plates, were evaluated through the determination of thickness, water wettability, roughness, and cross-cut adhesion tests. The antimicrobial activity of the iron amino-functionalized materials was also tested, as proof of concept, to monitor the growth of some bacterial strains.

## 2. Materials and Methods

### 2.1. Materials and Paint Formulation Synthetic Procedures

Plates of DH36 high-strength structural steel, conforming to the ASTM A131/A131M-19 standard [34] or to the equivalent grade ABS DH36, were selected as the substrate for depositing and testing the coatings.

The chemical composition limits, according to ASTM A131/A131M-19 Designation are shown in Table 1.

Silica and titania were functionalized with aminopropyl groups adopting a grafting method. In a typical procedure, a mixture of 1.00 g of calcined silica or titania in 15.0 mL of absolute ethanol and 1.00 mL of 3-aminopropyl triethoxysilane (APTES) was refluxed overnight. The materials were filtered in vacuo, washed with ethanol, and dried overnight at 120 °C. Iron (5% *w*/*w*) was supported by a wet impregnation procedure. In a typical procedure, iron nitrate nonahydrate (Fe(NO_3_)_3_·9H_2_O, 543 mg) was dissolved in 10 mL of water and added to aminopropyl silica or titania (1.5 g). The mixture was stirred overnight at room temperature, then it was filtered and washed with water.

Plates of DH36 high-strength structural steel, conforming to ASTM standards or the equivalent grade ABS DH36, were selected as the substrate for depositing and testing the coatings.

The preparation of paint formulation was carried out by mixing the Fe-based powder with a commercially available two-component polyamine-cured pure epoxy coating (Safeguard Universal ES, Jotun, “tie-coat, Jotun Italia Srl, Muggia, Trieste, Italy). Component A (6 g, Safeguard Universal ES Comp. A), component B (1.2 g, Safeguard Universal ES Comp. B), the diluent (0.36 g, Thinner n° 17), and SiO_2_-NH_2_-Fe or TiO_2_-NH_2_-Fe solid (75.6 mg) were mechanically mixed into a PP vessel by using a flat brush until a homogeneous paint was obtained. The as-obtained SiO_2_- or TiO_2_-based formulations were deposited on the surface of a metal plate (structural steel DH36 150 × 75 mm, thickness 5 mm) with a flat brush. The day before the application of the Fe-based formulation, a pristine metal specimen bearing a primer coating was treated with a tie coat paint as the basic surface (Jotun, Sandefjord, Norway) and dried overnight at room temperature.

Table 2 lists the four types of materials used in this study, with the code, description, and details.

**Figure 1:**
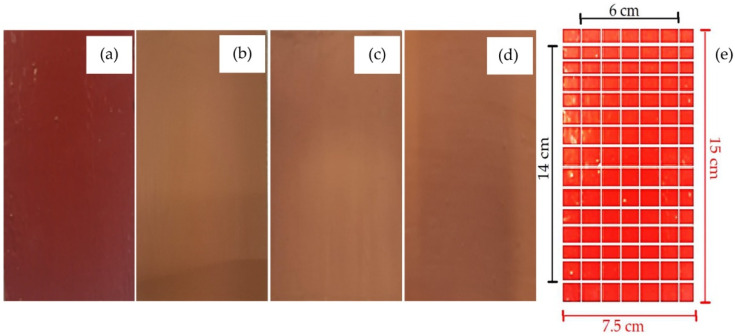
Materials of this study: C-Top (**a**), C-Tie (**b**), Si-Top (**c**), and Ti-Top (**d**) after their deposition on the structural steel DH36 with the internal measuring grid for the detection of thickness maps (6 cm × 14 cm): red colour bars refer to external geometry and black colour bars refers to the internal grid (**e**).

Figure 1a–d show the four coatings of this study after the deposition on the structural steel DH36 plate (geometry of 150 × 75 mm, thickness 5 mm); each picture geometry is specified in Figure 1e (red colour bars). Each coated structural steel plate was marked with a pencil that marked the various points to create an internal grid (14 cm × 6 cm; see the black colour bars in Figure 1e). Thickness measurements were taken on these grid points to create a mapping extended to the entire sample (see Figure 1e where the intersection point of the vertical white lines with the horizontal white lines, highlighted on the red substrate, defines the exact point where the thickness value is measured that will be reported in the thickness maps discussed in Section 3).

### 2.2. Chemical Characterization

The textural properties of the materials were examined with a Micromeritics ASAP2020 Plus 1.03 (Micromeritics, Ottawa, ON, Canada). The specific surface areas of the samples were evaluated through the analysis of the N_2_ adsorption isotherm at 77 K by using the BET method in the standard pressure range of 0.05–0.3 p/p0. The total pore volume, Vp, was calculated from the amount of nitrogen adsorbed at a relative pressure of 0.998, whereas mesopore size distribution values and mesopore volumes were estimated by applying the BJH model in the range of p/p0 of 0.1–0.98.

The thermogravimetric analysis of the samples was carried out in air using the TGA 1 Star System of Mettler Toledo (Mettler Toledo, Schwerzenbach, Switzerland). About 10 mg of sample was heated from room temperature to 100 °C, left at this temperature for 30 min, and then heated to 1000 °C at a rate of 10 °C/min in 40 mL/min of air.

The X-ray photoelectron spectroscopy (XPS) analyses were performed with a VG Microtech ESCA 3000 Multilab (VG Microtech/VG Scientific, East Grinstead, West Sussex, UK), equipped with a dual Mg/Al anode. Unmonochromatic Al Ka radiation (1486.6 eV) was used as the excitation source. The sample powders were mounted on a double-sided adhesive tape. The pressure in the analysis chamber was in the range of 10–8 Torr during data collection. The constant charging of the samples was removed by referencing all the energies to the C 1 s binding energy set at 285.1 eV. Analyses of the peaks were performed with the CasaXPS 2.3.26 software.

### 2.3. Physical/Mechanical Characterization

The four coatings were physically and mechanically characterized by thickness evaluation, wettability tests, and cross-section tests (adhesion strength evaluation before, at t = 0 days, and after, at t = 30 days, a period of immersion in seawater).

To measure the thickness of metal specimens deposited with coatings, a digital thickness gauge SA-MA Tools SA8850 (SAMA Italia, Viareggio, Italy) was utilized. A grid (14 cm × 6 cm; see the black color values in Figure 1e) was created on the metal specimen to acquire, at the intersection points, the different thickness values for the whole specimen. Thickness measurements were taken at each grid point by positioning the probe perpendicular to the specimen. To give a chromatic idea of the distribution of thickness values of the analyzed samples, chromatographic maps were created using a 14 × 6 matrix with the graphic processing software Origin © 2.019. The average of all the measurements was subsequently calculated.

The measurements of surface roughness were carried out using the portable and compact Surftest SJ-210 Series 178 roughness tester (Mitutoyo S.r.l., Milan, Italy), according to Equation:

$$R_{a}=\frac{1}{N}\sum_{i=1}^{n}\left|\mathrm{Yi}\right|$$

where R_a_ denotes the arithmetic mean of the absolute values of the deviations of the evaluation profile (Yi) from the mean line. The data presented are the average values from nine measurements for each type of sample.

The Wenzel contact angle (θ_w_) and that of Young (θ_y_, not dependent on the roughness) were assessed using the θ/2 method (prototype instrument—Engineering Department, Messina University) by measuring the contact angle of a 1 μL drop of deionized water placed on the horizontal surface of the sample, in accordance with ASTM D7334 [35] and Equation:

$$\begin{array}{cc} \theta_{w}=2arctg\;\left(\frac{2h}{d}\right); & \theta_{y}=arcos\;\left(\frac{cos\;\theta_{w}}{r}\right) \end{array}$$

where (d) is the diameter (in mm) and (h) is the height (in mm) of the drop. The data presented are the average values obtained from ten measurements for each sample [36].

**Figure 2:**
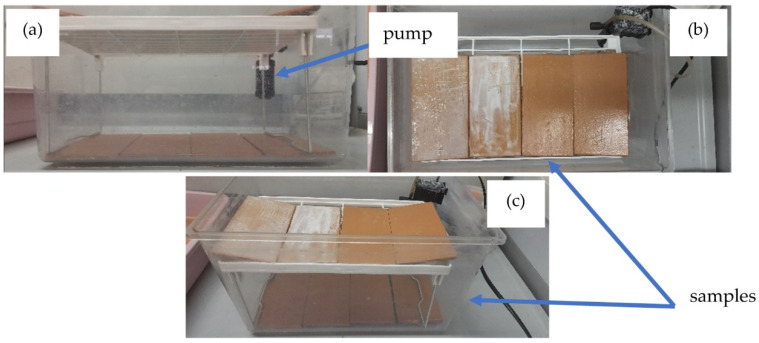
Different views of the set-up for the measurement of the resistance to seawater of the coatings deposited on structural steel plate samples and fully immersed in seawater: low side (**a**), top (**b**), high side (**c**).

The adhesion resistance of antifouling coatings was evaluated in seawater in accordance with ASTM D870-97 standards [37]. The samples were placed in aquarium tanks (rectangular shape of 30 × 20 cm, height of 20.3 cm) and completely submerged in seawater. To replicate the movement of seawater, a submerged aquarium pump (flow rate Q = 200 L/h, pipe diameter = 8 mm, flow velocity exiting the pipe = 1.11 m/s, flow velocity along the cross section = 0.0009 m/s) with a filter was utilized to ensure constant water recirculation over the surfaces. The water temperature was maintained at 24 °C, while the ambient temperature was kept at 23 °C. The seawater was refreshed every two weeks (Figure 2).

The samples were subsequently examined for potential bubble formation, softening, and loss of adhesion using the cross-cut test (Cross Hatch Adhesion Tester by Sama Tools—SADT502-5, SAMA Italia, Viareggio, Italy) before and after immersion in seawater after 1 month.

**Figure 3:**
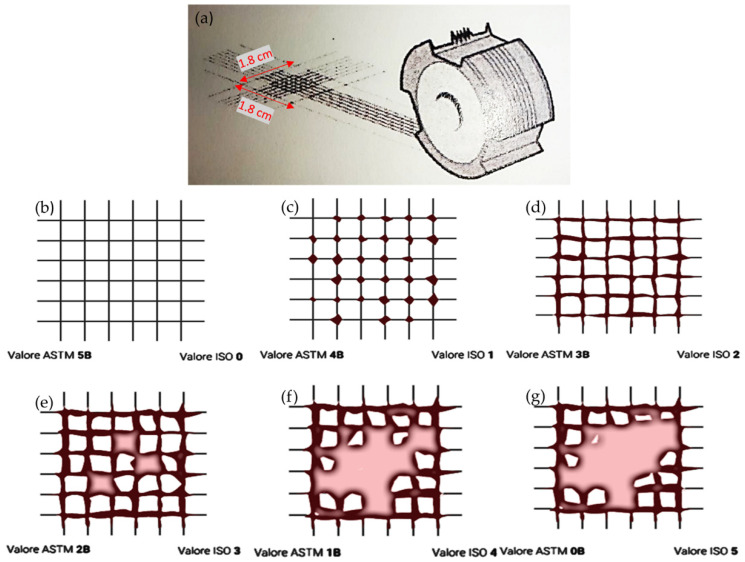
Geometry of a square-shaped grid generated during the cross-cut test (**a**) and ASTM/ISO standards: 5B/0 (**b**), 4B/1 (**c**), 3B/2 (**d**), 2B/3 (**e**), 1B/4 (**f**), 0B/5 (**g**) detachment types.

This method employs a suitable cutting tool with a blade space of 2 mm (for coatings of 60–120 microns) to score the coating down to the substrate. Specifically, on a 15 × 7.5 cm substrate, two cuts are made that intersect at 90 degrees, creating square-shaped grid patterns with four intersections on the substrate of 1.8 × 1.8 cm (see Figure 3a). The cross-cut area is then examined for any adhesion issues. The relevant standards for this test are D 3359-09e2 [38] and ISO 2409:2007 [39] (see Figure 3b–g).

### 2.4. Microtox Assay

Microtox^®^ toxicity tests were carried out on SiO_2_-NH_2_-Fe and TiO_2_-NH_2_-Fe materials. The Microtox^®^ toxicity tests were conducted using the luminescent bacterium *Vibrio fisheri* according to the standard procedures of the EN12457 protocol with the following modifications. The values of bioluminescence inhibition (indirect index of toxicity values) were reported as the effective concentrations (100 mg L^−1^) of toxicants resulting in a 50% decrease in bioluminescence (EC50). The EC50 with 95% confidence intervals was calculated, after 15 and 30 min of exposition, following the procedures outlined in the Microtox^®^ System Operating Manual (Microtox, Columbus, OH, USA, 2003). Each biocidal sample was compared with a reference un-toxicity matrix.

### 2.5. Bacteria, Culture Conditions, and Microbiological Tests

Microbial strains: three bacterial strains, namely, *Pseudoalteromonas* sp., *Alteromonas* sp., and *Pseudomonas* sp., were used in this study. All three were isolated from the marine environment and maintained in the culture collection of the Institute of Biological Resources and Marine Biotechnology (IRBIM)-CNR of Messina (Messina, Italy).

Microbial preparation: starter cultures were carried out by inoculating microbial cells into 10 mL of Marine Broth (MB; Difco, Milan) mineral medium. Cultures were grown in a rotary shaker (New Brunswick C24KC, Edison, NJ, USA; 150 rpm) at 20 ± 1 °C for 5 days. Mid-exponential-phase grown cells were harvested by centrifugation at 10.000× *g* for 10 min, washed twice with sterile MB, and inoculated into different 100 mL sterile Erlenmeyer flasks each containing 50 mL of Marine Broth.

Evaluation of the biocidal activity of the new functionalized coatings: mid-exponential-phase grown cells were harvested a second time as previously described and inoculated at a final concentration of 0.1 of optical density (OD600 nm) in a sterile medium supplemented with 100 mg L^−1^ of SiO_2_-NH_2_-Fe and TiO_2_-NH_2_-Fe materials. The cultures were incubated at 20 ± 1 °C for 9 days, with shaking (150× *g*, New Brunswick C24KC, Edison, NJ, USA). All experiments were carried out in triplicate. At the beginning of experimentation and regular intervals (3 days), the growth (biomass variations) of the cultures in this study was routinely assessed indirectly by measuring the turbidity (OD600 nm) using a UV–visible spectrophotometer (Shimadzu UV-160, Markham, ON, Canada).

## 3. Results and Discussion

**Scheme 1:**
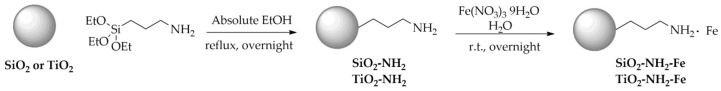
Synthesis of SiO_2_-NH_2_-Fe and TiO_2_-NH_2_-Fe.

The synthesis of Fe-based powders, as reported in Scheme 1, was carried out through the covalent grafting of (3-aminopropyl)triethoxysilane (APTES) onto the surface of commercial SiO_2_ or TiO_2_ in ethanol. The obtained hybrid materials SiO_2_-NH_2_ and TiO_2_-NH_2_ were used as supports for the immobilization of iron sites, which was performed by a wet impregnation procedure using Fe(NO_3_)_3_·9H_2_O as metal precursor. The final solids were labeled as SiO_2_-NH_2_-Fe and TiO_2_-NH_2_-Fe.

**Figure 4:**
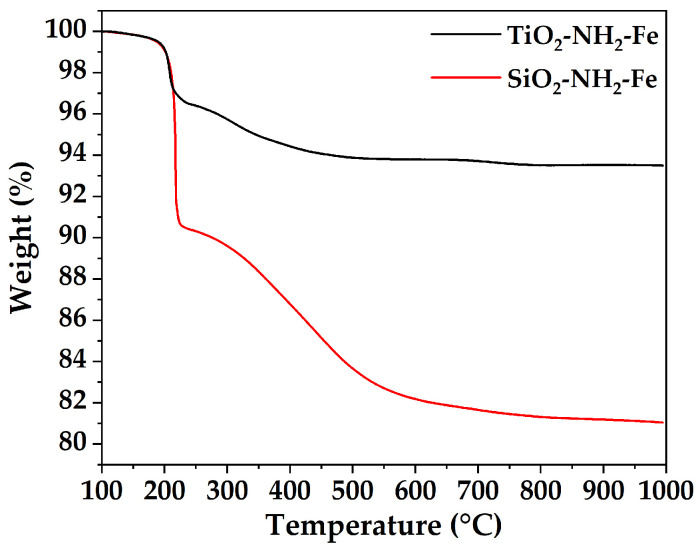
TGA profiles of SiO_2_-NH_2_-Fe and TiO_2_-NH_2_-Fe.

The thermogravimetric analysis (TGA) of SiO_2_-NH_2_-Fe and TiO_2_-NH_2_-Fe showed, in both cases, good thermal stability at 180 °C (Figure 4).

**Figure 5:**
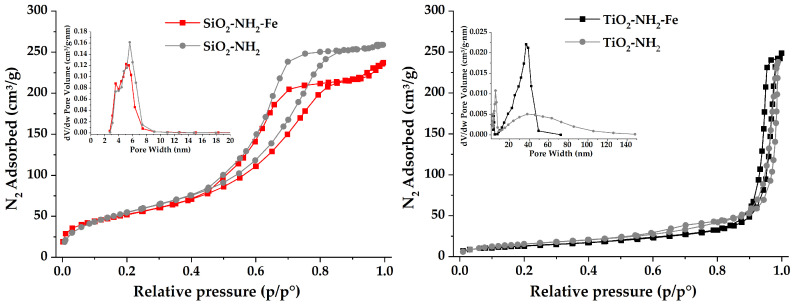
N_2_ adsorption/desorption isotherms of SiO_2_-NH_2_-Fe, SiO_2_-NH_2_-Fe, TiO_2_-NH_2_, and TiO_2_-NH_2_-Fe.

The higher organic loading of SiO_2_-NH_2_-Fe can be ascribed to the specific surface area of pristine SiO_2_ (SSA_BET_ 545 m^2^/g) when compared to bare TiO_2_ (SSA_BET_ 56 m^2^/g). The specific surface area value of silica implies a good -OH availability that is pivotal for the condensation of APTES functional groups. N_2_ physisorption measurements (Figure 5) were carried out on SiO_2_-NH_2_ (SSA_BET_ 208 m^2^/g), SiO_2_-NH_2_-Fe (SSA_BET_ 190 m^2^/g), TiO_2_-NH_2_ (SSA_BET_ 59 m^2^/g), and TiO_2_-NH_2_-Fe (SSA_BET_ 48 m^2^/g).

**Figure 6:**
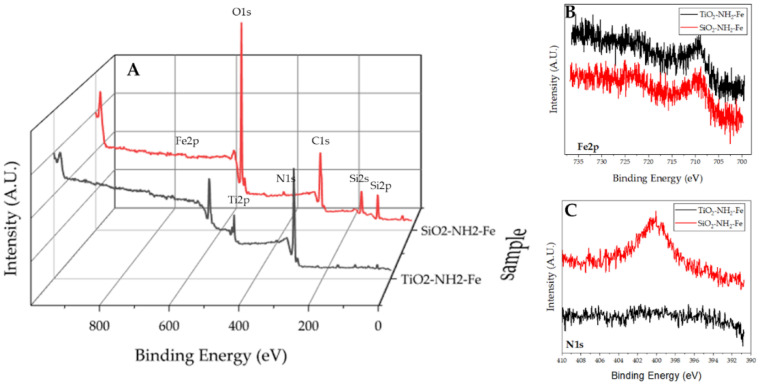
XPS spectra of SiO_2_-NH_2_-Fe and TiO_2_-NH_2_-Fe: (**A**) survey spectra; (**B**) Fe2p region; and (**C**) N1s region.

The XPS analysis was performed to analyze the surface of the synthesized additives. The survey spectra (see Figure 6A) show for both materials the peaks due to the bare supports. In addition, for SiO_2_-NH_2_-Fe, it is possible to appreciate the N1s peak due to the presence of the propylNH_2_ functionalization. The presence of Fe is not evidenced in the survey spectra. In order to better analyze the materials, the single regions (Fe2p and N1s) were recorded and the results are shown in Figure 6B,C, respectively. The presence of iron is confirmed by the presence of the characteristic Fe2p doublet in both materials. The low intensity of the peaks and the natural complexity of the Fe2p emission [40] make it difficult to evaluate the oxidation state of iron; however, the position and the shape of the profile are consistent with the iron in the +3 oxidation state. The analysis of the N1s region, showing the presence of a peak at 399.8 eV typical of the amino group [33], confirms that the surface of SiO_2_-NH_2_-Fe has been functionalized. By contrast, the absence of a visible N1s-related peak in the TiO_2_-NH_2_-Fe scan indicates that, in this sample, the functionalization was less effective.

Regarding the ecotoxicological assays, the Microtox tests show that SiO_2_-NH_2_-Fe and TiO_2_-NH_2_-Fe samples do not cause significant bioluminescence decay in *Vibrio fischeri* (Table 3). In the EN12457 protocol, the Microtox^®^ bioluminescent assay assessed in water typically shows an underestimated sensitivity to highly hydrophobic chemicals. This is primarily because these compounds have extremely low solubility in water and almost irreversible adsorption of matrix-like sediments.

**Figure 7:**
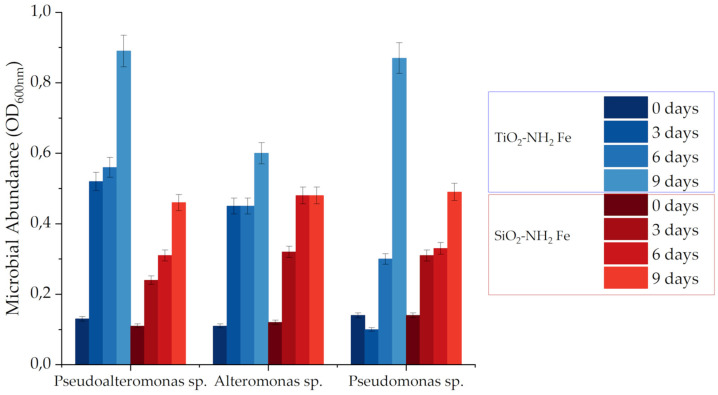
Growth of bacterial strains, measured as optical density (OD_600 nm_), in the presence of SiO_2_-NH_2_-Fe and TiO_2_-NH_2_-Fe. Error bar (5%).

The growth curves of the bacterial strains were monitored by measuring the optical density (OD) at 600 nm in the presence of two matrices: SiO_2_-NH_2_-Fe and TiO_2_-NH_2_-Fe. Measurements were taken over 9 days, with assessments every 3 days (Figure 7).

Strains grown with SiO_2_-NH_2_-Fe showed similar growth patterns initially, with the *Alteromonas* sp. strain peaking at day 6 before stabilizing. *Pseudoalteromonas* sp. also followed this trend, peaking at day 9, while *Pseudomonas* sp. exhibited the highest growth at the end. In contrast, the TiO_2_-NH_2_-Fe environment yielded higher growth for both *Pseudoalteromonas* and Pseudomonas strains. *Pseudoalteromonas* sp. reached values of 0.5 to 0.6 on days 3 and 6, then increased to about 0.9 by day 9. *Pseudomonas* sp. showed fluctuating growth initially but also increased to approximately 0.9 on day 9. The *Alteromonas* strain grew from 0.1 on day 0 to 0.6 on day 9.

In summary, bacterial strains demonstrated better growth in the TiO_2_-NH_2_-Fe suspension compared to SiO_2_-NH_2_-Fe.

These preliminary tests assessed the antimicrobial activity of the iron amino-functionalized materials and highlighted their promising properties.

The antibacterial mechanism of Fe(III) against bacteria was studied by Sun et al., who reported that bacteria could adsorb Fe(III) and reduce it to Fe(II) [22].

As noted by Videira-Quintela et al. [41], the antibacterial effect can be related to Fe(II) ions, which produce reactive oxygen species (ROS) including hydroxyl radicals (OH·), leading to bacterial damage and growth inhibition [42,43]. Additionally, hydroxyl radical production likely occurs via the Fenton reaction: Fe(II) + H_2_O_2_ → Fe(III) + OH + OH^−^, where Fe(II) reacts with hydrogen peroxide (H_2_O_2_), generated through the dismutation of superoxide (2O_2_^−^ + 2H^+^ → H_2_O_2_ + O_2_) during Fe(II) autoxidation [44].

The antibacterial effect of TiO_2_ nanoparticles modified with APTES is documented by Rokicka-Konieczna [30], whereas we developed corresponding functional materials based on SiO_2_ and TiO_2_ with enhanced functional iron-based sites.

The antibacterial action of SiO_2_ nanoparticles operates through various mechanisms, such as adsorption and membrane disruption, the generation of ROS, and intracellular penetration. These processes work in concert to enhance the bactericidal effect of SiO_2_ nanoparticles as proof of their promise of antimicrobial activity [45].

The enhanced antibacterial activity of SiO_2_-NH_2_-Fe compared to TiO_2_-NH_2_-Fe could be attributed to both the higher loading of amino groups in SiO_2_-NH_2_-Fe and the greater specific surface area of silica, which facilitates better adsorption onto bacterial cell surfaces. The adsorption process involves electrostatic interactions between the hydroxyl groups and the bacterial membrane, as well as between the amino groups [31] and the bacterial membrane. Upon adsorption, the designed nanomaterials interact with the lipid bilayer of the bacterial membrane, disrupting its integrity and structure. This damage increases membrane permeability, causing leakage of essential cellular components like ions and cytoplasmic molecules. The loss of these vital elements disrupts the bacterial cells’ homeostasis and function, ultimately resulting in cell death [45].

Functional additives were mechanically dispersed in a commercial tie coat to be applied on the surface of metal prototype plates by using a flat brush, according to the description in Section 2.

The average thickness values are listed in Table 4. The value of the commercial tie coat and topcoat was 498 microns and 515 microns, respectively. As expected, the thickness value improved after the addition of the two topcoats (653 microns (Si-Top) and 674 microns (Ti-Top)).

**Figure 8:**
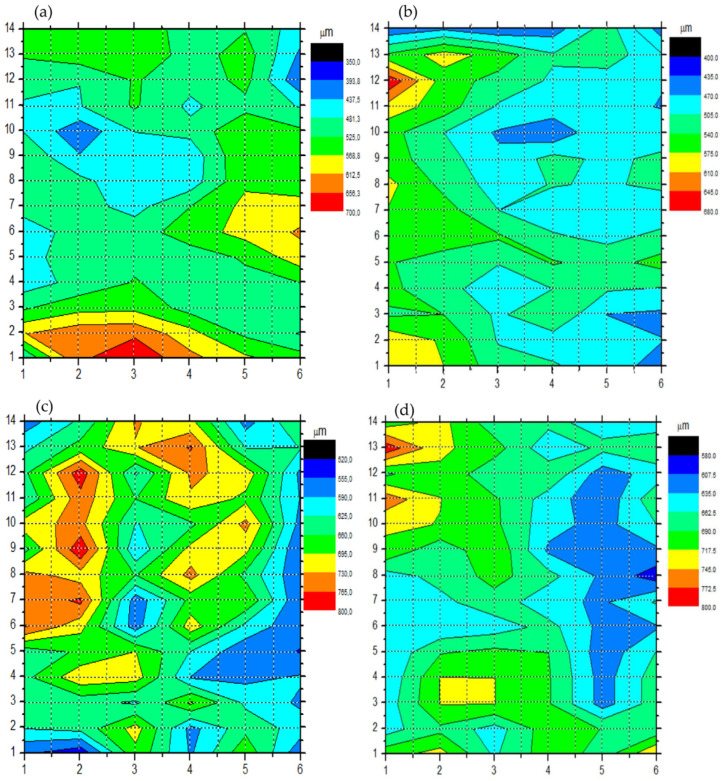
Thickness maps of C-Tie-498 μm (**a**), C-Top 515 μm (**b**), Si-Top 653 μm (**c**), and Ti-Top 674 μm (**d**).

Figure 8 shows the thickness distribution map calculated on the 6 cm × 14 cm surface of the metal plate.

Generally, all the maps reveal a thickness distribution that is not always homogeneous. There is a greater inhomogeneity in the Si-Top compared to the other topcoats. This is due to the manual deposition of the coatings.

**Figure 9:**
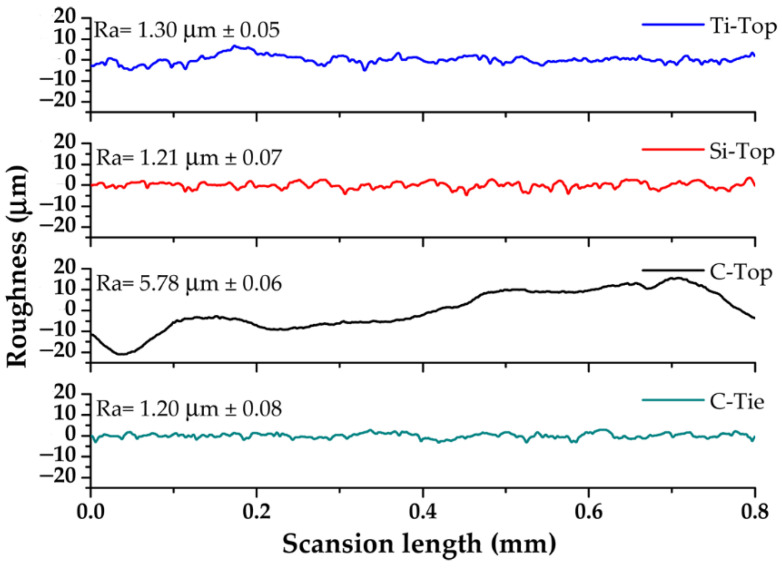
Typical roughness profiles of the substrates of the coatings deposited on metal.

Table 4 summarizes the roughness Ra values of the coatings. Figure 9 shows that the C-Top coating is much rougher (R_a_ = 5.78 μm) than the other substrates.

From the graph below and the values detailed in Table 4, it can be seen how the contact angle θ_w_ (°) and θ_y_ (°/μm) of the coatings, except for C-Top, which is hydrophilic (θ_w_ = 73°, θ_y_ = 87°), have hydrophobic characteristics, that is, they have a contact angle greater than 90°.

**Figure 10:**
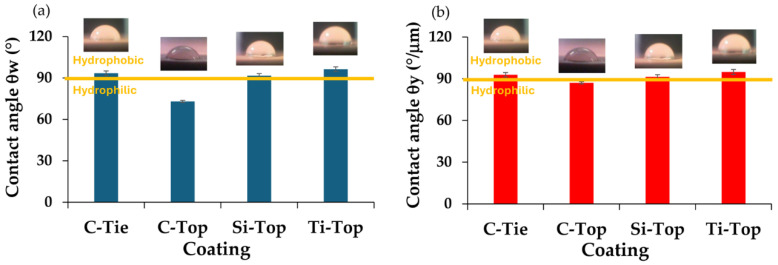
Contact angle θ_w_ (**a**) and θ_y_ (**b**) of different sample coatings.

In particular, Ti-Top exhibits the highest contact angle value, resulting in the more hydrophobic coatings among the four investigated (Figure 10).

**Figure 11:**
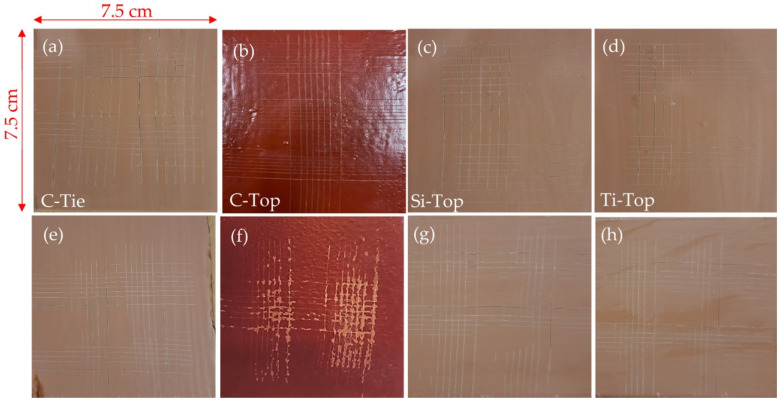
Cross-cut test results of the coatings: (**a**) C-Tie; (**b**) C-Top; (**c**) Si-Top; (**d**) Ti-Top; and after 1 month of immersion in seawater: (**e**) C-Tie; (**f**) C-Top; (**g**) Si-Top; (**h**) Ti-Top.

The cross-cut test (refer to Figure 11) showed that three coatings—C-Tie, C-Top, and Si-Top—exhibited the highest adhesion rating of 5B/0, according to the evaluation scale presented in Figure 3, before undergoing the seawater resistance test. In contrast, the Ti-Top coating had a lower adhesion rating of 4B/1.

The high adhesion value (5B/0) of the C-Tie, C-Top, and Si-Top coating agrees with the finding of other authors who studied similar epoxy-based coatings containing ZnO-APTES, and they proved that the adhesin power was the highest [46,47].

After 30 days of testing in seawater, the adhesive capacity of all coatings decreases. The commercial topcoat demonstrates the lowest resistance among all the coatings analyzed because it drops from a value of 5B/0 to a value of 2B/3 (see Figure 3).

The most resistant coating, Si-Top, decreases from a value of 5B/0 to 4B/1. In comparison, the Ti-Top coating, which is less resistant, drops further from a value of 4B/1 to 3B/2. Therefore, the Si-Top antifouling coating is the best option regarding adhesive capacity and is also less hydrophobic than the Ti-Top coating.

The enhanced performance of Si-Top, along with its increased hydrophilicity, can be attributed to the higher concentration of amino groups in the SiO_2_-based nanopowder with respect to the titania-supported one. The increased loading of functional groups is associated with the large surface area of the SiO_2_ support, which, in turn, results in a higher density of -OH groups, further enhancing the hydrophilic properties.

Furthermore, manual deposition involves human error due to the operator, his/her manual skills, and his/her experience in carrying out manual deposition work. In our work, the coatings were always deposited by the same operator and under the same temperature and pressure conditions. That is, the operator carried out the deposition in an extremely short time to contain any variables, and using the same type of brush (flat).

Although we did not obtain a deposition that was entirely homogeneous, as can be seen from the images in Figure 8 (due to understandable human error), our coatings still managed to adhere well to the metal substrate, as demonstrated by the results of the mechanical tests (Table 4). Therefore, the yield would certainly be increased if a deposition were to be carried out with more precise technologies (for example, spray coating), further improving the already good performance of our coatings.

## 4. Conclusions

This article focused on novel coatings developed to protect DH 36 steel plates widely used in offshore structures and shipbuilding. SiO_2_ and TiO_2_ were modified with amino groups to create hybrid materials for immobilizing iron sites. These were incorporated into paint formulations, which were applied to steel prototype plates. The coatings’ properties, including thickness, water wettability, roughness, and adhesion, were tested, along with their performance in seawater degradation, and microbiological tests.

The main findings were as follows:

- SiO_2_-NH_2_-Fe powders exhibited better antibacterial performance compared to TiO_2_-NH_2_-Fe powders, making SiO_2_-NH_2_-Fe a more suitable candidate for antifouling applications;
- The higher adhesion and lower surface roughness exhibited by the SiO_2_-based material compared to the TiO_2_-based material, together with its hydrophobicity (θ > 90°), further support its potential to improve the durability and performance of coatings in marine environments;
- The application of a primer coating followed by a tie coat containing TiO_2_-NH_2_-Fe or SiO_2_-NH_2_-Fe powders on DH 36 steel plates eliminates the need for an additional topcoat, presenting a notable strategic advantage for simplifying the coating process while maintaining effective steel surface protection.

## Figures and Tables

**Table 1:** Composition limits for ASTM DH36 high-strength structural steel (weight %).

C	Mn	Si	P	S	Al	Cb (Nb)	Ti	Cu	Cr	Ni	Mo	Ca	Fe
0.18	0.9–0.16	0.1–0.5	0.035	0.035	0.015	0.02–0.05	0.02	0.35	0.20	0.40	0.08	0.05	Bal.

**Table 2:** Codes and materials of this study.

Code	Typology of Material	Description	Details
C-Top	Sea Quantum Ultra S	Commercial topcoat	Cutting-edge, single-component antifouling with silyl acrylate chemical reaction
C-Tie	Safeguard Universal ES, Jotun “tie-coat”	Commercial tie coat	Two-component epoxy-vinyl with polyamide hardener (5:1)
Si-Top	Si-Tie + SiO_2_-NH_2_-Fe	Commercial Tiecoat + our SiO_2_-based additive	
Ti-Top	Ti-Tie + TiO_2_-NH_2_-Fe	Commercial Tiecoat + our TiO_2_-based additive	

**Table 3:** Measurement of Microtox^®^ toxicity on SiO_2_-NH_2_-Fe and TiO_2_-NH_2_-Fe materials.

Sample	Concentration (mg L^−1^)	Matrix	EC50 mg L^−1^ (15’)	EC50 mg L^−1^ (30’)
SiO_2_-NH_2_-Fe	100	Powder	>100	>100
TiO_2_-NH_2_-Fe	100	Powder	>100	>100

**Table 4:** Thickness, roughness, wettability, and adhesion power of all coatings.

Sample Code	Thickness [μm]	Wenzel/Roughness/Young Contact Angle			Cross-Cut Test ASTM D 3359-09e2/ISO2409:2007	
		θ_w_ [°]	Ra [μm]	θ_y_ [°/μm]	(t = 0 days)	(t = 30 days)
C-Tie	498 ± 96	93.47 ± 1.62	1.20 ± 0.08	92.89 ± 1.62	5B/0	4B/1
C-Top	515 ± 52	73.04 ± 0.73	5.78 ± 0.06	87.11 ± 0.73	5B/0	2B/3
Si-Top	653 ± 73	91.65 ± 1.58	1.21 ± 0.07	91.36 ± 1.58	5B/0	4B/1
Ti-Top	674 ± 64	96.37 ± 1.75	1.30 ± 0.05	94.90 ± 1.75	4B/1	3B/2

## Data Availability

The data presented in this study are available on request from the corresponding authors.
